# Compressing neonatal-perinatal medicine fellowship training: a critical appraisal of the American Board of Pediatrics proposed 2-year pathway

**DOI:** 10.1038/s41372-026-02743-5

**Published:** 2026-06-03

**Authors:** Brooke Vergales, Melissa Scala, Christie Bruno, Cynthia Crabtree, Megan Gray, Suma Hoffman, Courtney McLean, Deirdre O’Reilly, Vilmaris Quinones Cardona, Erynn M. Bergner, Jennifer M. Brady, Lindsay Johnston, Patrick Myers, Misty Good, Patrick J. McNamara, Sara DeMauro, John Loyd, Jill Maron, Camilia R. Martin, Steven J. McElroy, Annemarie Stroustrup, Sarah N. Taylor, Trent E. Tipple, Ravi M. Patel, Shetal Shah, Clara Song, Alexis Davis, Munish Gupta, Marilyn Escobedo, Heather French

**Affiliations:** 1 Northwell Health; Cohen Children’s Medical Center and the Zucker School of Medicine at Hofstra, New Hyde Park, NY USA; 2 Stanford University School of Medicine, Palo Alto, CA USA; 3 Yale University School of Medicine, New Haven, CT USA; 4 University of Louisville School of Medicine, Norton Children’s Hospital, Louisville, KY USA; 5 University of Washington School of Medicine, Seattle, WA USA; 6 Children’s National Hospital, George Washington School of Medicine, Washington, DC USA; 7 University of Nebraska Medical Center, Omaha, NE USA; 8 University of Vermont College of Medicine, Burlington, VT USA; 9 Drexel University School of Medicine, Philadelphia, PA USA; 10 University of Oklahoma College of Medicine, Oklahoma City, OK USA; 11 University of Cincinnati School of Medicine, Cincinnati, OH USA; 12 Northwestern University Feinberg School of Medicine, Chicago, IL USA; 13 University of North Carolina School of Medicine, Chapel Hill, NC USA; 14 University of Iowa Stead Family Children’s Hospital, Iowa City, IO USA; 15 Perelman School of Medicine at the University of Pennsylvania, Philadelphia, PA USA; 16 University of Texas at Austin Dell School of Medicine, Austin, TX USA; 17 The Warren Alpert School of Medicine at Brown University, Providence, RI USA; 18 Weill Cornell Medicine, New York, NY USA; 19 University of California Davis School of Medicine, Sacramento, CA USA; 20 Emory School of Medicine, Atlanta, GA USA; 21 Maria Fareri Children’s Hospital, New York Medical College, Valhalla, NY USA; 22 Southern California Permanente Medical Group, Anaheim, CA USA; 23 Beth Israel Deaconess Medical Center, Harvard Medical School, Boston, MA USA

**Keywords:** Scientific community, Health occupations

## Abstract

The American Board of Pediatrics (ABP) has proposed a competency-based fellowship model offering a 2-year clinical pathway with 18 training blocks and no scholarly requirement. This position statement, representing neonatal-perinatal medicine (NPM) training program directors, division leaders, and key stakeholders, contends that the model is not viable for NPM training. Compressing 18 clinical blocks into 24 months would require approximately 3500 annual training hours, nearly double a sustainable NICU workload, approaching Accreditation Council for Graduate Medical Education duty-hour limits, while reducing didactic education by one-third. Survey data from 110 of 111 NPM programs show 76% opposition. Concerns include trainee wellness, reduction in program complement, workforce and funding implications, and the erosion of scholarly training essential to advancing neonatal care. We propose alternatives, including a 2-year residency + 3-year fellowship (2 + 3) pathway and restructured residency tracks, addressing competency gaps while preserving training quality, duration, and collaborative governance.

## Problem statement

The American Board of Pediatrics (ABP) recently announced a new competency-based fellowship model that introduces two distinct pathways: a clinically focused 2-year pathway with an optional third year dedicated to scholarship, research, or advanced training without a scholarly work product requirement. In parallel, clinical training requirements were increased approximately 50%, from 12 months to 18 clinical blocks (usually defined as weeks) over a condensed 2-year period, with the earliest implementation in July 2028 [1].

While we recognize and support innovation in fellowship training, we are deeply concerned by the substance of this proposal, the process by which it was developed, and the potential ramifications for our trainees and our specialty. Existing literature raises significant questions about readiness for independent practice after two years of training. A study by Pitts et al. [2] suggests that pediatric subspecialty fellows are not adequately prepared for unsupervised practice after 2 years, while others highlight that graduates of current 3-year programs often require continued supervision in at least one core competency domain [3, 4]. These findings underscore the need for caution when adjusting fellowship duration, as they may lead to serious harm to graduate competency, workforce quality, and patient care in neonatal-perinatal medicine (NPM).

Equally concerning is the ABP’s limited engagement of physician educators and speciality leaders in shaping this redesign. Key stakeholders, including the Organization of Neonatal-Perinatal Medicine Training Program Directors (ONTPD), the community dedicated to implementing training models for future neonatologists, and the ABP Neonatal-Perinatal Medicine Sub-Board, were not meaningfully included in this decision-making process. This highlights a broader structural concern: hospital-based pediatric subspecialties, such as NPM, remain proportionally underrepresented on national pediatric executive committees [5–9], which limits incorporation of the specific needs of critical care training.

In addition, limited transparency regarding the rationale for these changes, inconsistent messaging on organizational processes, and an accelerated rollout without clear logistical frameworks have constrained opportunities for collaboration. Together, these factors risk eroding trust and may have unintended consequences for trainee preparedness. At a time when the training is already under strain, this proposal may further challenge the development of future neonatologists, with potential implications for care quality and ongoing scientific advancement [10].

NPM is uniquely complex. Neonatologists provide care for critically ill premature infants and newborns, many with complex congenital conditions, requiring mastery of advanced therapies such as mechanical ventilation, inhaled nitric oxide, therapeutic hypothermia, surgical co-management, extracorporeal membrane oxygenation (ECMO) support, neonatal transport, and delivery room resuscitation at the limits of viability. Our trainees must also master the changing fetal and infant physiology, biology, and pathophysiology across all organ systems, using a comprehensive, holistic approach. In addition, neonatologists lead multidisciplinary teams, guide families through high-stakes and emotionally charged decisions, and navigate ethically complex care, including end-of-life transitions.

Compounding these challenges, reductions in intensive care unit (ICU) exposure during pediatric residency over the last two decades have significantly limited incoming fellows’ baseline knowledge and experience. Currently, fellows may begin training with as little as one month of neonatal ICU (NICU) exposure, minimal experience with in-house calls or level IV NICU care, and few procedural opportunities [11]. As a result, fellowship programs must increasingly build foundational competencies from the ground up [12], further reinforcing the need for additional training time, rather than less.

The ICU environment is also physically and emotionally demanding. Burnout remains a significant concern, with one study estimating that up to one-third of neonatologists experience burnout [13]. Within this context, the expectation that NPM fellows complete 18 four-week blocks of intensive clinical training within 24 months is neither feasible nor responsible. Such a model would likely violate ACGME duty-hour requirements or necessitate workarounds that increase fatigue, compromise well-being and patient safety, and elevate the risks of burnout, attrition, and professionalism concerns [14, 15]. Further, over 40,000 residents and fellows across the United States are members of the Committee of Interns and Residents (CIR), a local of the Service Employees International Union (SEIU). Institutions with unionized trainees will face significant challenges in implementation. They will need to navigate the complexities of the newly proposed competency model put forth by the ABP through collective bargaining.

While the ABP has suggested flexibility in what may count toward clinical training, we emphasize that alternative experiences cannot replace the essential value of core NICU service time, including overnight and longitudinal care. These experiences are foundational to developing and refining clinical judgment, procedural competence, prioritization and communication skills, and the resilience and maturity required for independent practice when performing under pressure in high-acuity environments.

For these reasons, a 2-year fellowship is not a viable model for NPM. While it may be appropriate for selected pediatric subspecialties, it cannot accommodate the breadth, intensity, and longitudinal complexity of neonatology training.

## Neonatal-perinatal medicine training program director perspectives

Neonatal program directors (PDs) and associate program directors were surveyed immediately after the ABP announced the proposed 2-year fellowship model (April 2026). A total of 148 responses were received, representing 110 of 111 programs. Among respondents, 76% opposed the two-year fellowship pathway, 13% were neutral, and only 11% supported it.

The top five concerns identified by members of the ONTPD with this model were: 1) insufficient time for competency development, 2) increased burnout due to compressed clinical training, 3) loss of individualized professional development, 4) diminished opportunities for research, quality improvement, and medical education innovation, and 5) an accelerated implementation timeline that limits the ability to anticipate and mitigate foreseeable and unforeseeable programmatic consequences. In contrast, support for this model focused on reduced scholarly work requirements, lower training costs, and the potential to enhance recruitment by shortening training duration.

Notably, NPM PD perspectives on broader structural change also shifted following the announcement. In Fall 2025, 59% of PDs favored a separate NPM Residency, a figure that increased to 75% following the ABP’s announcement. Similarly, support for separating NPM from general pediatrics as a department rose from 43% to 63% between Fall 2025 and April 2026, suggesting growing concern about the adequacy of current and proposed training paradigms and erosion of the relationship between general pediatrics and neonatology [16].

## Current training models vs proposed new model

Under the current training structure, pediatric residents have limited ICU exposure. Current ACGME requirements include 12 weeks of ICU training (only 4 weeks are required in the NICU) and 4 weeks of newborn nursery, totaling approximately 1000 clinical hours of ICU and newborn care [11]. Procedural competency requirements during residency are minimal and may be met through simulation; as a result, many graduating residents lack proficiency in essential NICU-specific procedures and real-life experience with neonatal resuscitation. Indeed, only a minority of graduating residents complete even one of all core pediatric procedures before graduation [17]. This underscores the critical importance of fellowship in achieving procedural and clinical competence, particularly as progression through training is strongly associated with increasing procedural success [18].

The current NPM fellowship training model requires a minimum of 12 months of clinical training within a three-year structure (Table 1). Published data from the ONTPD indicate that fellows complete a median of 13 four-week blocks (52 weeks; IQR 48, 54) of NICU daytime service and 150 night shifts or calls (IQR 120, 165) over three years [19]. The most common additional rotations include a clinical orientation at the beginning of fellowship and 2-4 weeks each in the cardiac ICU, maternal-fetal medicine or fetal centers, and the neonatal follow-up clinic. Educational conferences contribute an additional average of 4 h per week and are supplemented by procedural bootcamps, simulation, point-of-care ultrasound, and structured communication training.

**Table 1:** NPM fellowship common program requirements comparison between current, proposed, and ABP models.

	Current Fellowship 3-year model - 156 weeks	NICU Proposed Fellowship 3-year model -156 weeks	ABP Proposed 2-year model - 104 weeks
Clinical experience^a^	52 weeks	72 weeks	72 weeks
Structured educational program in clinical/basic sciences^b^	115 h/3 yrs	115 h/3 yrs	77 h/2 yrs
Active fellow planning/participation in NPM conferences and M&M conferences^c^	108 h/3 yrs	108 h/3 yrs	72 h/2 yrs
Education related to pregnancy and the fetusd	295 h/3 yrs	295 h/3 yrs	245 h/2 yrs
Bioethics training	30 h/3 yrs	30 h/3 yrs	20 h/2 yrs
Health care economics and high-value care	70 h/3 yrs	70 h/3 yrs	50 h/2 yrs
Scholarly experience	Minimum of 12 months	Area of concentration minimum of 12 months	Not required

^a^Clinical experience includes NICU rotations, delivery room experience, high-risk neonatal follow-up, and supervision of residents.

^b^Must include anatomy, physiology, biochemistry, embryology, pathology, microbiology, pharmacology, immunology, genetics, nutrition/metabolism, maternal physiology and adaptation to pregnancy.

^c^Must include M&M and case conferences.

^d^Must include maternal physiological, biochemical, and pharmacological influences on the fetus, fetal physiology, fetal development, placental function, physiological and biochemical adaptations to birth, cellular, molecular, and developmental biology and pathology relevant to diseases of the neonate, psychology of pregnancy and maternal-infant interaction; breastfeeding and lactation, growth and nutrition, and genetics.

Estimates based on French et al. Educational Landscape of Neonatal-Perinatal Medicine Fellowship Programs [22].

**Fig. 1:**
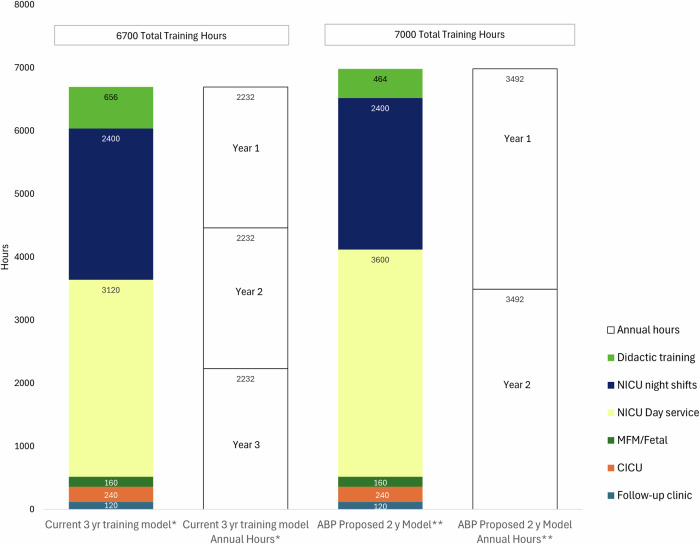
Comparison of Fellowship Clinical Training Hours: Current and ABP Proposed Training Model. Based on current median reported clinical training across NPM programs, we present an example clinical program to meet ABP model targets while maintaining current night shift volume of 150 night shifts/calls during training. Assumptions: 1 block = 4 weeks, NICU service week = 60 h, Night shift = 16 h, CICU week = 60 h, MFM and follow-up clinic week = 40 h, didactics = 4 h/week × 48 weeks per year + 80 h of Orientation.

Using this data, the current NPM fellowship training model provides an estimated 6700 total hours of clinical and didactic education over three years, or approximately 2230 h annually (Fig. 1). While this already exceeds the proposed sustainable workload range for NICU physicians (1600–1900 h annually) [20], it is distributed across a longer time frame that allows for progressive clinical skill acquisition, ongoing procedural exposure, and professional development, with adequate time for recovery between clinical shifts [20, 21].

The ABP asserts that current NPM fellowship graduates are not meeting the competencies necessary for autonomous practice and has proposed that fellows complete 18 clinical education blocks. As NPM programs define the length of a clinical education block as 4 weeks, per ACGME block diagram requirement [11], 18 educational blocks are equivalent to 72 weeks of training distributed between the NICU and other relevant training rotations. Even under a conservative estimate that preserves current educational elements (including night shifts) and excludes time spent on scholarly projects, this would increase the total clinical training from 6700 to 7000 h (Fig. 1) [22]. When compressed into a two-year framework, fellows would be required to complete approximately 3500 h of training per year—nearly double the upper threshold for a sustainable workload for ICU physicians [20, 21].

Operationally, this translates to an average of 80 h of work per week, after accounting for the required 4 weeks of annual vacation. Such a model approaches or exceeds ACGME duty hour limits and raises serious concerns about trainee well-being, patient safety, and the integrity of the educational experience. The proposed compression fundamentally shifts fellowship from the existing model of progressive competency development to one of unsustainable maximal workload, without evidence that such intensity improves readiness for unsupervised practice.

The proposed 2-year training model would have a direct and underappreciated impact on program complement, the number of fellows a program is accredited to train at any given time. Under the current three-year fellowship model, most NPM fellowship programs structure training across 13 four-week clinical rotations per year [11], allowing a predictable and sustainable distribution of fellows across clinical environments. The proposed model requires a 38% increase in clinical density. Because clinical training environments, including the NICU, can accommodate only a fixed number of trainees on service at any given time, this compression creates an immediate scheduling problem: more training blocks must be completed in less time within a space with a fixed capacity. Procedural opportunities are a particularly limited training resource and a critical rate-limiting element in trainee volume. As a result, programs may be forced to reduce their complement if they cannot provide sufficient clinical and procedural experience At a time when the pediatric subspecialty workforce is already under strain, a structural reduction in the number of fellows that can be trained at any given time would directly undermine the goal of producing an appropriate supply of competently trained neonatologists.

## Trainee wellness and education impacts

Under the proposed 2-year fellowship model, NPM fellows will work an average of 78 clinical hours/week, a 59% increase from the 49 h per week required under the current 3-year model. This estimate already excludes time for scholarly activity and assumes 4 weeks of vacation. Critically, most of these hours are concentrated within the NICU, a clinical environment defined by high cognitive load, procedural complexity, and significant physical and emotional demands on trainees.

The ABP has indicated that the increase in clinical educational blocks need not occur exclusively in the ICU. However, if neonatology fellows are not demonstrating readiness for unsupervised practice at graduation, a deficiency the ABP itself has acknowledged, [2–4] programs will have little choice but to concentrate additional training time in the NICU, the primary environment in which graduates will ultimately practice. The continued decline in resident ICU exposure exacerbates this. The ABP cannot simultaneously cite inadequate clinical readiness as a justification for this model and suggest that remediation can occur outside the very setting where that readiness must be demonstrated.

Recent data highlight an already strained trainee workforce. In the 2024-2025 ACGME Well-Being survey, 36% of NPM fellows reported feeling emotionally drained at work, 40% reported requiring more time than in the past to decompress, and 43% felt worn out after work [23]. A compressed training model will exacerbate these trends, with increased risk of burnout, attrition, and compromised patient safety. The increased clinical burden also leaves minimal time to address personal health needs, and any parental, medical, or family leave would inevitably prolong training.

Although a shorter fellowship may initially appear appealing, trainees may underestimate its physical and psychological demands, particularly given limited ICU exposure during residency. Fellowship training requires not only the acquisition of knowledge and skills but also time for recovery, reflection, reading to support didactic and experiential learning, and the formation of professional identity. Experiential learning theory emphasizes reflection as essential to transforming experience into lasting competency [24]. A schedule dominated by sustained high-intensity clinical work leaves insufficient time for this reflective practice, undermining adult learning and long-term retention, and risks deterring qualified candidates from pursuing neonatology altogether.

Beyond individual well-being, compressing training into a shorter timeframe disrupts the longitudinal maturation essential to neonatology. Clinical expertise in the NICU depends on repeated exposure to complex, evolving clinical courses, nuanced decision-making, and procedural competence built over time. This cannot be accomplished effectively in a 2-year time frame. Equally important is the development of leadership skills – guiding multidisciplinary teams, mentoring junior trainees, and building unit-wide trust – a key component of a neonatologist’s professional identity.

Current NPM fellowship didactic curricula are structured to cover all 17 domains outlined by the ABP, with individual fellowship programs dedicating approximately 4 h per week to protected didactic education, totaling roughly 600 h over the course of 3 years. Compressing this curriculum into 2 years would create compounding problems: first, the higher clinical burden under the proposed model would inevitably increase missed educational sessions due to fatigue and duty-hour constraints; and second, programs would be forced to reduce both the depth and breadth of content. The result is a training model that simultaneously demands more of fellows clinically while delivering less to them educationally.

## Funding concerns

Fellowship training in NPM is supported by Medicare, Medicaid, and the Children’s Hospital Graduate Medical Education (CHGME) program, which constitute the primary federal sources of graduate medical education (GME) funding in the United States. These funding streams are structurally linked to training positions and duration of training, such that changes in fellowship length directly affect institutional reimbursement and program sustainability. National policy analyses have demonstrated that GME financing is not neutral, but actively shapes institutional decision-making regarding program size, training structure, and workforce distribution [16, 25].

In this context, shortening fellowship training may introduce financial incentives for institutions – particularly children’s hospitals that rely heavily on CHGME due to limited Medicare reimbursement – to restructure program length based on fiscal considerations rather than educational competency. This could also undermine the feasibility of an optional third year of scholarship or extending training for fellows who require additional time to achieve readiness for independent practice.

## NPM division directors perspectives

The ABP proposal raises concerns about the future of academic neonatology. Over the past fifty years, outcomes for extremely preterm infants and those with complex diseases have improved dramatically. Neonatal mortality (deaths per 1000 live births) fell from 11.6 in 1975 to 3.6 in 2022 [26]. These improvements would not have been possible without a commitment to scientific discovery, innovation, and the translation of discovery into clinical care. Compression of training and the removal of scholarly expectations risk losing future neonatologist-scientists and weakening the critical relationship between clinical care and scientific advancement.

The proposed fellowship changes were discussed at the Association of Academic Neonatology Division Directors (AANDD) meeting on April 24, 2026. While there was support for competency-based training, there was opposition to a 2-year fellowship model. Concerns centered on the compression of 18 clinical training blocks into 24 months, the loss of meaningful scholarly activity, and the downstream impact on trainees’ ability to critically evaluate the literature and apply evidence to their clinical practice. AANDD agreed that many graduating fellows are not fully prepared for independent practice, which reinforces the need for sufficient training time, not less. Finally, there was a strong consensus that a 2-year fellowship would not provide the breadth and depth of training needed for an academic career, particularly at the early-career stage, where scholarly contributions are essential for academic promotion.

## Proposed solutions

### Re-evaluation

Neonatal-specific stakeholders, including program directors, division directors and researchers, must partner and convene with national organizations to discuss and achieve consensus on optimum pathways for neonatology fellowship training. Implementation of a 2-year Neonatal-Perinatal Medicine pathway should be deferred until further discussion and broader stakeholder input can be obtained. Further, any newly proposed design of fellowship training must be pilot-tested and rigorously evaluated prior to widespread dissemination to uncover unforeseen issues in implementation.

### Reimagining the scholarly work requirement

Reassessment of scholarly work product requirements is essential given the myriad ways neonatologists can perform scholarly work; however, eliminating or minimizing this component erodes a defining feature of pediatric subspecialty training. Every neonatal fellow should engage in meaningful professional development through a focused area of concentration.

Potential areas of concentration or individualized professional development pathways could include research/physician-scientist development, medical education, quality improvement & patient safety, advocacy, ethics and palliative care, or clinical concentration (i.e. bronchopulmonary dysplasia, ECMO, hemodynamics, neurocritical care, cardiac critical care, nutrition, and point-of-care ultrasound).

### A re-envisioned training model: the 2 + 3 pathway model

**Fig. 2:**
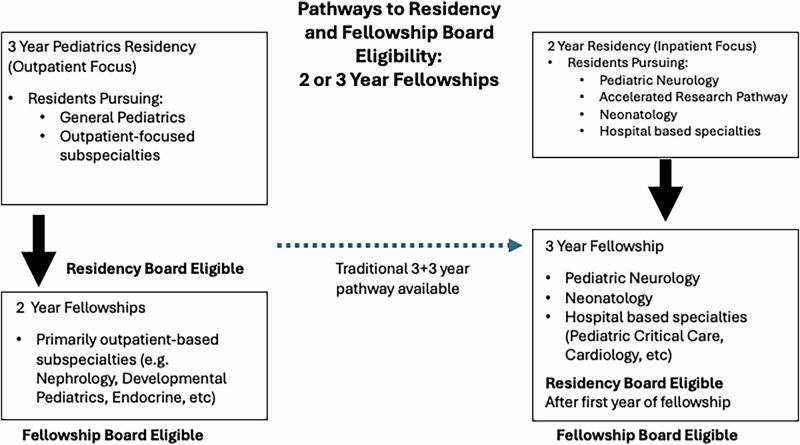
Proposed pathways for pediatric residents based on clinical interest and/or career path.

We strongly advocate that 3 years remain the standard for NPM fellowship training, consistent with existing recommendations [27]. However, reducing total training time is possible through a restructured 2 + 3 model: 2 years of pediatric residency followed by 3 years of NPM fellowship (Fig. 2).

This model would require a genuine partnership with the ABP, the ACGME, and pediatric residency programs to shorten the residency requirement to 2 years of inpatient-focused pediatric training and 3 years of subspecialty training. Key features include:

#### Early identification pathway

Residents who identify a strong interest in neonatology or other hospital-based specialties as a PGY-1 could enter a dedicated, inpatient-focused training track.

#### Depth over breadth in residency

A 2-year pediatric residency would emphasize hospital-based medicine, procedural competency, and early ICU immersion, replacing the traditional 3-year breadth of training and eliminating rotations without clear relevance to future intensivists. Some core requirements of pediatric training, such as mental health training, could be shifted to a fellowship where they can be most effectively trained to meet the particular needs of their patient populations.

#### Dual certification

Graduates would remain eligible for the general pediatrics board certification exam after the 3rd year of training, as well as for the NPM certification exam after completing 5 total years of training.

#### Parallel traditional pathway

The current 3-year pediatrics residency and 3-year fellowship pathway would remain available to those who decide to pursue a neonatology fellowship later in, or after, completion of residency training.

#### Safeguards

Anticipated safeguards during a shortened residency training period include competency-based medical education verification, residency program director approval, and early and longitudinal mentorship with ICU faculty.

#### Scalability across subspecialties

The 2 + 3 model could be co-developed with other interested hospital-based pediatric subspecialties (e.g., Cardiology, Pediatric Critical Care, Gastroenterology) as a common pathway into hospital-based or critical care fellowships. Additionally, the two-core pediatric years model has already been successfully implemented in both the Pediatric Neurology and Accelerated Research Pathway tracks.

An alternative approach is to continue the existing 3 + 3 (3 years of residency and 3 years of fellowship) model while pursuing targeted reforms at the residency level in partnership with the ACGME. Specifically, we propose restructuring pediatric residency training into two distinct tracks: an outpatient and general pediatrics track for residents pursuing primary care or outpatient subspecialties, and an inpatient and critical care track for residents pursuing hospital-based subspecialties such as NPM. In a three-year in-patient residency focused, we would propose increasing ICU time from 12 to 20 weeks while decreasing ambulatory care to 32 weeks. This reallocation would ensure residents entering inpatient subspecialty fellowships arrive with a stronger foundation in hospital-based clinical decision-making, and procedural skills, as well as the beginning of their professional identity formation.

Rather than compressing fellowship training at the expense of educational depth, the 2 + 3 model and the reimagined 3 + 3 model both address the competency concerns the ABP has identified at their root,in residency, while preserving the time and environment that fellowship training requires to produce graduates who are genuinely ready for unsupervised practice.

A final, more complex, and therefore least preferred option is the creation of an independent training and certification program, separate from the historical home of neonatology within the ABP. Ultimately, the goal of the neonatology community is the effective, safe, and sustainable training of the next generation of neonatologists, the best care of neonates, and innovations in neonatal science that translate into improved clinical outcomes. We are prepared to achieve this goal in any way possible.

## Closing statement

The compression of increasingly complex ICU training into a shortened timeframe without meaningful partnership with educational and academic leaders in neonatology risks patient outcomes, trainee well-being, physician preparedness, and the future of neonatology. The neonatology community is prepared with pragmatic, data-informed solutions; however, sustainable progress requires collaborative design rather than top-down implementation. We therefore urge reconsideration of the current ABP 2-year clinical fellowship proposal and implementation timeline to allow for a deliberate, collaborative redesign process that ensures training reform is thoughtful, feasible, and aligned with the needs of trainees, educators, and the patients they will serve.
